# ﻿Three new species of *Psilogamasus* Athias-Henriot, 1969 (Acari, Parasitiformes, Parasitidae) from Southwest China

**DOI:** 10.3897/zookeys.1259.171008

**Published:** 2025-11-12

**Authors:** Mao-Yuan Yao, Jian-Xin Chen, You-Fang Wu, Rong Ren, Tian-Ci Yi, Dao-Chao Jin

**Affiliations:** 1 College of Agriculture, Anshun University, Anshun, China; 2 Provincial Special Key Laboratory for Development and Utilization of Insect Resources of Guizhou, Guizhou University, Guiyang, China; 3 Institute of Entomology, Guizhou University, Guiyang, China

**Keywords:** Decomposing leaves, description, key, mites, new species, Parasitinae, taxonomy

## Abstract

The present study reports three new species of *Psilogamasus*. Among them, one species, *Psilogamasus brachysaccatus* Jin & Yao, **sp. nov.** from the Xizang Autonomous Region, China, was described and illustrated based on adult females and males. Two species, *Psilogamasus decemtrichus* Ren & Yi, **sp. nov.** and *Psilogamasus bidens* Ren & Yi, **sp. nov.** from Yunnan Province, China, were described and illustrated based on females. *Parasitus truncatus* Tseng is transferred from the genus *Parasitus* to *Psilogamasus*. A key to the known species of *Psilogamasus* is provided.

## ﻿Introduction

The family Parasitidae comprises more than 500 species and 47 genera in two subfamilies, Parasitinae Oudemans, 1901 and Pergamasinae Juvara-Bals, 1972 (Hrúzová and Fenďa 2018; Makarova 2019; Juvara-Bals 2019; Yao et al. 2020, 2024). The genus *Psilogamasus*, belonging to the subfamily Parasitinae, was erected by Athias-Henriot (1969) with *Psilogamasus hurlbutti* Athias-Henriot, 1969 as its type species. It was a monotypic genus when first described. The genus *Taiwanoparasitus* was erected by Tseng in 1995 with *Taiwanoparasitus pentasetosus* as its type species. Hrúzová and Fenďa (2018) transferred two species from the Chinese mainland, *Vulgarogamasus brachysternalis* Ma & Lin, 2005 and *V. longascidiformis* Ma & Lin, 2005, to *Taiwanoparasitus* (Tseng 1995; Ma and Lin 2005). Recently, Yao et al. (2020) indicated that *Taiwanoparasitus* is newly synonymized with *Psilogamasus*, and four species from the Chinese mainland, namely *T. pentasetosusT. brachysternalis*, *T. longascidiformis*, *Vulgarogamasus lingulatus* Bai & Ma, 2013, have been transferred to *Psilogamasus*.

To date, six species of *Psilogamasus*, *P. brachysternalis* (based on female), *P. hurlbutti* (female and male), *P. lingulatus* (female), *P. longascidiformis* (female), *P. pentasetosus* Tseng, 1995 (female), and *P. pentatideus* Yao & Jin, 2020 (female and male), have been described worldwide (Yao et al. 2020). Except for *P. hurlbutti* which has been recorded in Tanzania and America, the mites of the genus have only been reported in China (Athias-Henriot 1969; Hennessey and Farrier 1989; Tseng 1995; Ma and Lin 2005; Bai and Ma 2013; Yao et al. 2020).

During a survey of Chinese Parasitidae, three new species of *Psilogamasus* were discovered. Herein, we describe these new species and provide an identification key to the known species of *Psilogamasus*.

## ﻿Material and methods

Mites were extracted from decomposing leaves by Berlese-Tullgren funnels for 12–24 hours and stored in 75% alcohol. Mites were cleared in Nesbitt’s solution and then mounted on slides in Hoyer’s medium. Specimens were observed and illustrated under a Nikon DS-Ri2 microscope, and figures were edited with Adobe Photoshop CC2021. All measurements are given in micrometers (μm).

The system of idiosomal setal nomenclature follows Hyatt (1980). Terminology for leg chaetotaxy follows Evans (1963a); palp chaetotaxy follows Evans (1963b); adenotaxy and poroidotaxy follow Athias-Henriot (1971, 1975); and measuring follows Yao et al. (2022).

## ﻿Results

### ﻿Family Parasitidae Oudemans, 1901

**Subfamily Parasitinae Oudemans, 1901**

#### ﻿Genus Psilogamasus

Taxon classificationAnimaliaMesostigmataParasitidae

Athias-Henriot, 1969

102ED992-F096-548D-AA90-0ECDE4B2A83B

##### Type species.

*Psilogamasus hurlbutti* Athias-Henriot, 1969

##### Diagnosis.

Both sexes. Dorsal idiosoma with less than 30 pairs of setae; setae *z5* of dorsal hexagon similar to *j5* and *j6* in form (smooth), while different in length (*z5* longer); tritosternum biramous; opisthogastric shield bearing five to six pairs of setae; setae *ZVI* and gland pores *gv2* absent (present in *P. decemtrichus* sp. nov.); seta *al* of palp femur comblike, *all* and *al2* of palp genu entire and spatulate distally; corniculi small; epistome trispinate.

Female with separated podonotal shield with 16–18 pairs of setae, and opisthonotal shield with five to six pairs of setae (ten pairs in *P. decemtrichus* sp. nov.); genital shield triangular or subtriangular; peritrematal shield anteriorly fused to podonotal shield and posteriorly free; movable digit of chelicerae with three or four teeth; setae *avl* and *av2* on femur II, *avl* on genu II and tibia II acicular.

Male with holodorsal shield and transverse suture in central region; movable digit of chelicera with five or six teeth; setae *avl* and *av2* on femur II modified into spurs fused at base; seta *avl* on genu II and tibia II each modified into spur.

#### ﻿ Psilogamasus brachysaccatus

Taxon classificationAnimaliaMesostigmataParasitidae

Yao & Jin sp. nov.

049A404C-F402-591C-BC0B-DFBACCB15EAC

https://zoobank.org/59A8B989-D6F9-491A-8FAB-4EEA5E6D0C7B

Figs 1, 2, 3, 4, 5

##### Diagnosis.

**Female.** Podonotal and opisthonotal shields with 18 and five pairs of setae, respectively; setae *j1* slightly pilose, *r3* simple; endogynium comprised of a short semi-circular sac with three to five small teeth on each side of the leading edge; opisthogastric shield bearing six pairs of setae; setae *ZV1* and gland pores *gv2* absent; Peritrematal groove extending anteriorly to beyond coxa II; movable digit of chelicera with four teeth.

**Male.** Seta *j1* slightly pilose; opisthogastric region with seven pairs of setae; central prong of gnathotectum serrated apically and lateral prongs acuminate distally; movable digit of chelicera with four teeth; dorsal seta on fixed digit of chelicera smooth; femur II with a main spur (proximal) and an axillary process (distal), genu II and tibia II each with a small spur (Fig. 5E). Other characteristics as in female.

##### Description.

**Female** (*N* = 4). Idiosoma weakly sclerotized, length 672–710, width 481–498.

**Figure 1.**
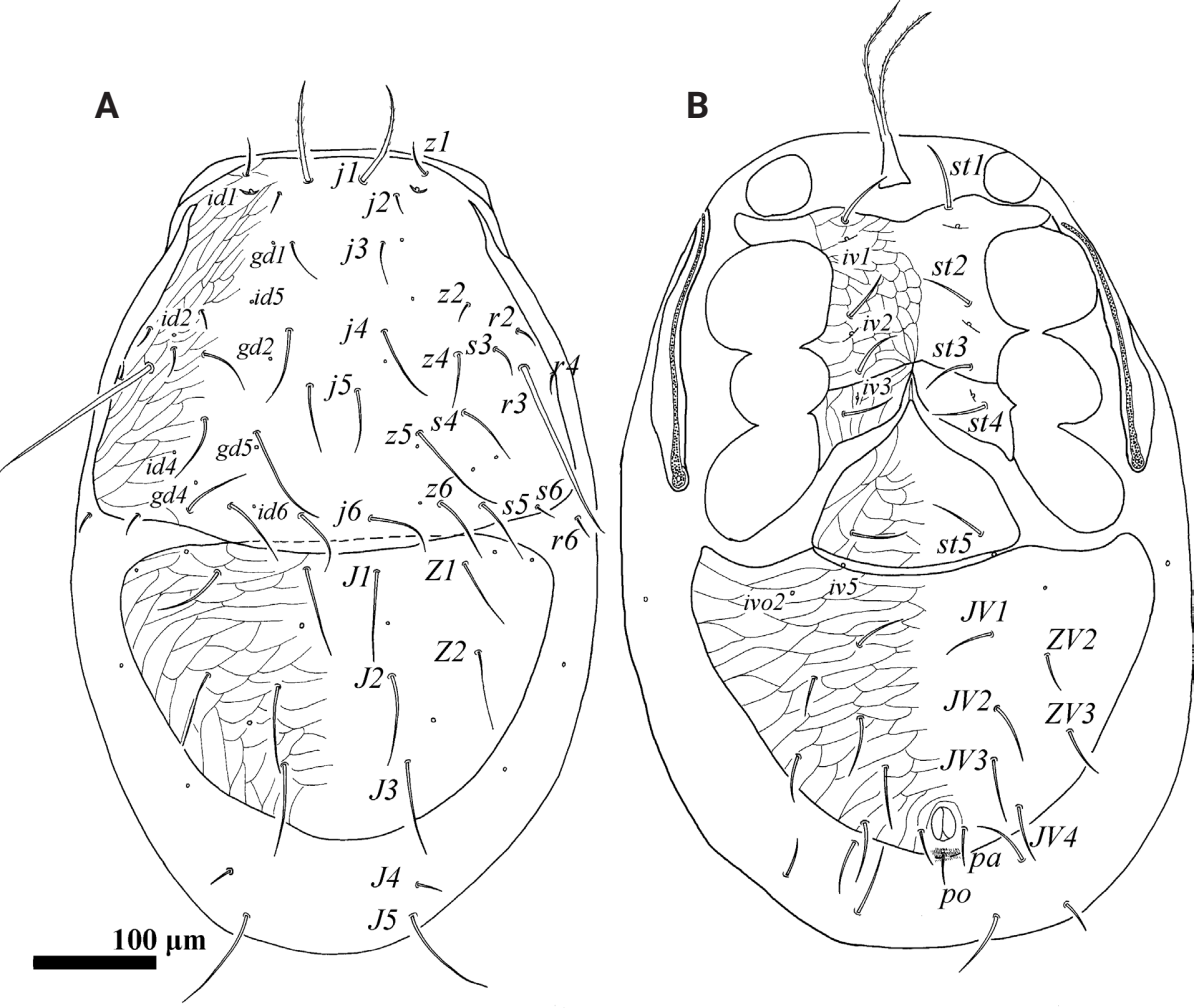
*Psilogamasus brachysaccatus* Yao & Jin, sp. nov., female. A. Dorsum; B. Venter.

***Dorsum*** (Fig. 1A). Podonotal and opisthonotal shields separated and with rough reticulation. Podonotal region with 19 pairs of setae, of which 18 pairs on podonotal shield and a pair (seta *r6*) off shield. Opisthonotal region bearing seven pairs of setae, of which *J1*–*J3* and *Z1*, *Z2* on opisthonotal shield, *J4* and *J5* off shield. Seta *j1* stout and slightly pilose, other dorsal setae smooth. Seta *r3* longer than others, setae *j2*, *j3*, *z1*, *z2*, *s3*, *s6*, *r2, r4*, *r6* and *J4* tiny and short. Lengths of dorsal setae: *j1* 74–78, *j2* 15–17, *j3* 32–35, *j4* 66–70, *j5* 58–62, *j6* 58–61, *z1* 26–28, *z2* 16–18, *z4* 47–50, *z5* 90–95, *z6* 57–62, *s3* 31–34, *s4* 58–61, *s5* 60–62, *s6* 15–17, *r2* 15–17, *r3* 159–165, *r4* 15–17, *r6* 15–17, *J1* 79–82, *J2* 78–82, *J3* 80–84, *J4* 16–17, *J5* 92–95, *Z1* 57–61, *Z2* 65–69.

**Figure 2.**
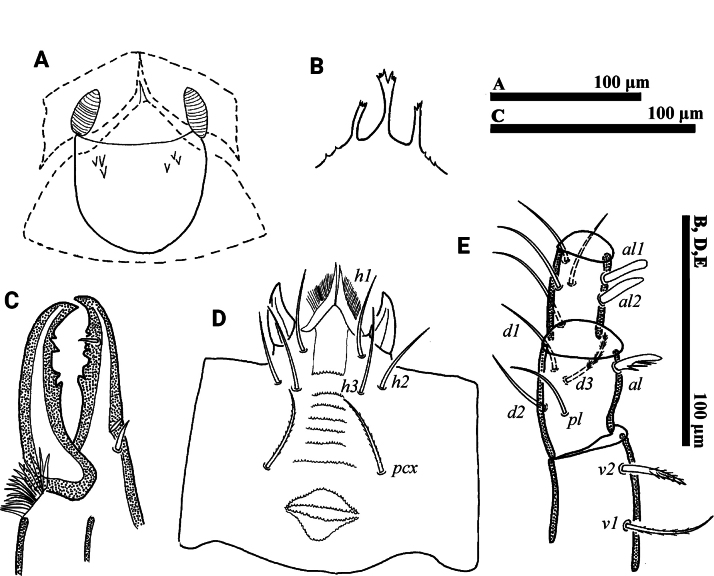
*Psilogamasus brachysaccatus* Yao & Jin, sp. nov., female. A. Endogynium; B. Gnathotectum; C. Chelicera; D. Subcapitulum; E. Trochanter, femur and genu of palp.

***Venter*** (Figs 1B, 2A). Tritosternum with two pilose laciniae (111–120) and a smooth base (42–45). Presternal platelets absent. Sternal shield reticulated, bearing three pairs of setae (*st1*–*st3*), *st1* (48–52) longer than *st2* (38–41) and *st3* (42–43). Metasternal shields separated from sternal shield by medially arched groove, bearing setae *st4* (39–42). Epigynial shield bearing setae *st5* (40–42). Anterior margin of epigynial shield with glassy angulation apex. Endogynium comprised of a short semi-circular sac with three to five small teeth on each side of the leading edge, and anterolaterally with two inner spindle-shaped structures underneath metasternal shields (Fig. 2A). Setae *ZV1* and gland pores *gv2* absent. Opisthogastric shield reticulate, bearing six pairs of setae (*JV1*–*JV4*, *ZV2* and *ZV3*). Setae *pa* and *po* equal in length (27–30). Peritrematal groove length 219–238, extending to anterior level of coxa II. Peritrematal shield fused with podonotal shield anteriorly and separated at level of seta *j2*. Opisthogastric soft cuticle with three pairs of setae. All ventral setae smooth. Lengths of setae on opisthogastric shield: *JV1* 42–45, *JV2* 46–49, *JV3* 47–49, *JV4* 47–50, *ZV2* 32–34, *ZV3* 42–43.

***Gnathosoma*** (Fig. 2B–E). Gnathotectum (Fig. 2B) with three prongs, serrated apically and emerging from base with one to three small teeth on each side. Movable digit of chelicera with four teeth, fixed digit with six teeth (Fig. 2C). Corniculus length 27–34, short and horn-shaped; deutosternal groove with 11 denticulate rows. Setae *h1*–*h3* smooth, *h1* 46–50, *h2* 40–43, *h3* 47–50 in length; *pcx* pilose, 48–52 in length (Fig. 2D). Palp length 228–239; trochanter with two pilose setae, *v1* stouter than *v2*; femur with five setae, of which *al* comblike, *d3* pilose; genu with six setae, of which *al1* and *al2* spatulate distally, remaining setae smooth. Trochanter, femur and genu of palp as in Fig. 2E.

**Figure 3.**
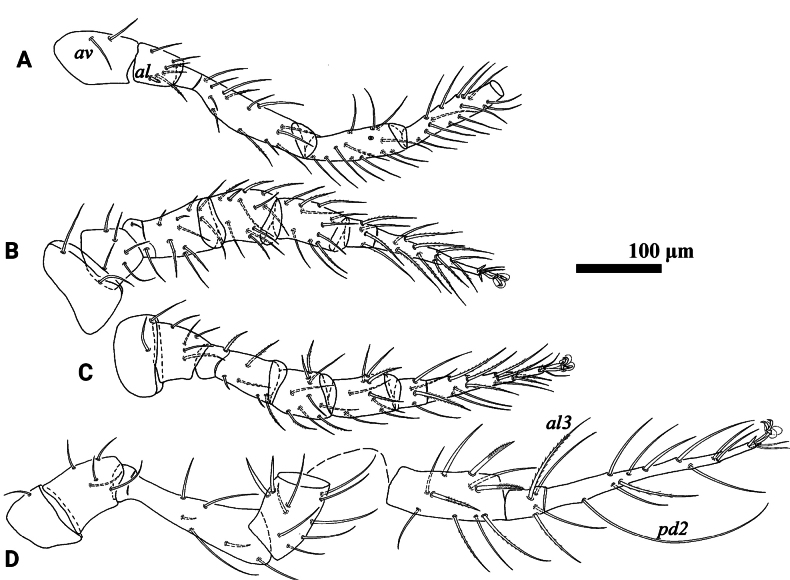
*Psilogamasus brachysaccatus* Yao & Jin, sp. nov., female. A. Coxa–tibia of leg I; B. Leg II; C. Leg III; D. Leg IV.

***Legs*** (Fig. 3). Lengths of legs I–IV: I 865–910, II 572–634, III 565–642, IV 907–990. Most leg setae smooth and setae on genu IV and tibia IV usually longer than others. Seta *al* on trochanter I stout and pilose. Most setae on tarsi II and tarsi III slightly pinnate. Setae *av1* and *pv1* on tarsi II–IV spur-like. Seta *pd2* (282–301) on tarsus IV longer than all other leg setae.

**Figure 4.**
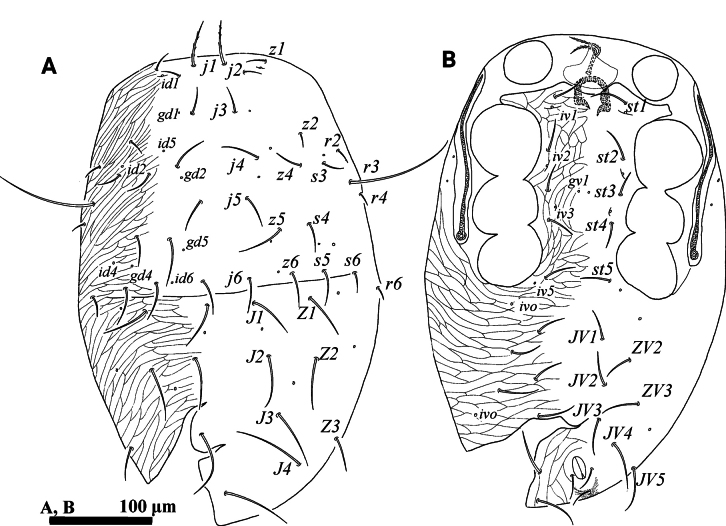
*Psilogamasus brachysaccatus* Yao & Jin, sp. nov., male. A. Dorsum; B. Venter.

**Figure 5.**
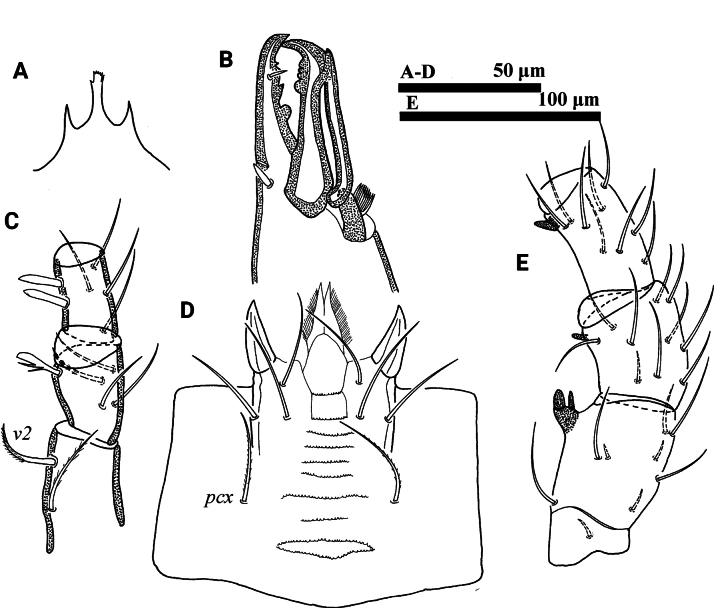
*Psilogamasus brachysaccatus* Yao & Jin, sp. nov., male. A. Gnathotectum; B. Chelicera; C. Trochanter, femur and genu of palp; D. Subcapitulum; E. Femur, genu and tibia of leg II.

**Male** (*N* = 3; Figs 4, 5) Idiosoma length 476–495, width 306–317.

***Dorsum*** (Fig. 4A). Reticulate holodorsal shield covering entire dorsum, with a suture closely anterior to setae *J1*, not reaching lateral margin of idiosoma. Podonotal region with 18 pairs of setae. Opisthonotal region bearing eight pairs of setae. All setae inserted on reticulate holodorsal shield. Seta *j1* slightly pilose, other dorsal setae smooth. Setae *j2*, *j3, z1*, *z2*, *s3*, *s6*, *r2*, *r4*, *r6* finer than others. Lengths of dorsal setae: *j1* 47–50, *j2* 17–19, *j3* 27–30, *j4* 40–44, *j5* 35–38, *j6* 35–39, *z1* 19–21, *z2* 16–19, *z4* 29–33, *z5* 50–55, *z6* 36–40, *s3* 24–26, *s4* 31–33, s5 35–39, *s6* 21–23, *r2* 18–20, *r3* 128–132, *r4* 14–15, *r6* 14–15, *J1* 48–53, *J2* 55–58, *J3* 63–67, *J4* 74–76, *Z1* 47–48, *Z2* 51–53, *Z3* 37–40.

***Venter*** (Fig. 4B). Tritosternum with two pilose laciniae (42–50), and a smooth base (17–20). Presternal shields absent. Genital lamina poorly visible, anteriorly extending to form a hyaline and smooth protrusion. Sternogenital shield bearing five pairs of setae (*st1*–*st5*). Anterior margin of sternogenital shield concave, posterior margin fused to opisthogastric region. Opisthogastric region with seven pairs of smooth setae. Setae *pa* and *po* equal in length (22–25). Peritrematal groove as in female, length 191–224. Lengths of setae on sternogenital region: *st1* 35–37, *st2* 32–34, *st3* 26–28, *st4* 26–28, *st5* 30–32, as well as on opisthogastric one: *JV1* 30–32, *JV2* 36–39, *JV3* 38–40, *JV4* 47–50, *JV5* 48–50, *ZV2* 32–34, *ZV3* 42–43.

***Gnathosoma*** (Fig. 5). Gnathotectum (Fig. 5A) with three prongs, central one serrated apically and lateral ones acuminate distally, emerging from nude base. Movable and fixed digit of chelicera with four teeth, respectively (Fig. 5B). Palp 189–205 in length, as in female, trochanter, femur and genu as shown in Fig. 5C. Subcapitulum (Fig. 5D) with setae *pcx* (30–31) pilose, *h1* (36–38), *h2* (32–35) and *h3* (40–43) smooth; deutosternal groove with ten rows, corniculus (23–25) and internal mala acute as in female.

***Legs***. Lengths of legs I–IV: I 799–812, II 531–544, III 512–539, IV 838–880. Leg II stouter than others. Femur II with a main spur (proximal) and an axillary process (distal), genu II and tibia II each with a small spur (Fig. 5E). Other characteristics as in female.

##### Other stages.

Unknown.

##### Etymology.

This species is named after its endogynium, which is composed of a short, semi-circular sac structure.

##### Material examined.

***Holotype*.** China • 1 ♀ (slide no. XZ2019071901), Xizang Autonomous Region, Linzhi City, Bome County; 29°54'33"N, 95°29'36"E; 2624 m a.s.l.; 19 Jun. 2019; collected from decomposing leaves by Jian-Xin Chen, ***Paratypes*.** China • 3 ♀ and 3 ♂ (XZ2019071902–XZ2019071902), the same data as the holotype.

The holotype and paratypes are deposited in the Institute of Entomology, Guizhou University, Guivang, China (GUGC).

##### Remarks.

The female of the newly described species is morphologically similar to *P. bidens* Ren & Yi, sp. nov. in the setal numbers on the podonotal, opisthonotal and opisthogastric shields, and the shape of seta *j1*. However, the female *P. brachysaccatus* Yao & Jin, sp. nov. is different from *P. bidens* Ren & Yi, sp. nov. as follows: (1) dorsal seta *r3* simple, vs. pilose in the latter; (2) presternal platelets absent, vs. a pair of presternal platelets present in the latter; (3) three prongs of gnathotectum each apically serrated and emerging from base with 1–3 small teeth on each side, vs. bifid and emerging from nude base in the latter; (4) endogynium with a semi-circular sac, vs. tongue-shaped in the latter; and (5) seta *v1* on palp trochanter pilose, vs. simple in the latter.

#### ﻿ Psilogamasus decemtrichus

Taxon classificationAnimaliaMesostigmataParasitidae

Ren & Yi sp. nov.

A97D579B-2ECB-53D6-A60A-AB7F6FE1C30E

https://zoobank.org/B627FA48-0110-410B-B562-379A6094572B

Figs 6, 7, 8

##### Diagnosis.

**Female.** Podonotal and opisthonotal shields with 18 and ten pairs of setae, respectively; setae *j1* and *r3* simple; endogynium tongue-shaped with some small teeth on each side of the leading edge; setae *ZV1* and gland pores *gv2* present; opisthogastric shield with six pairs of setae; Peritrematal groove extending anteriorly to beyond coxa II; movable digit of chelicera with three teeth.

##### Description.

**Female** (*N* = 5). Idiosoma weakly sclerotized, length 428–468, width 259–278.

**Figure 6.**
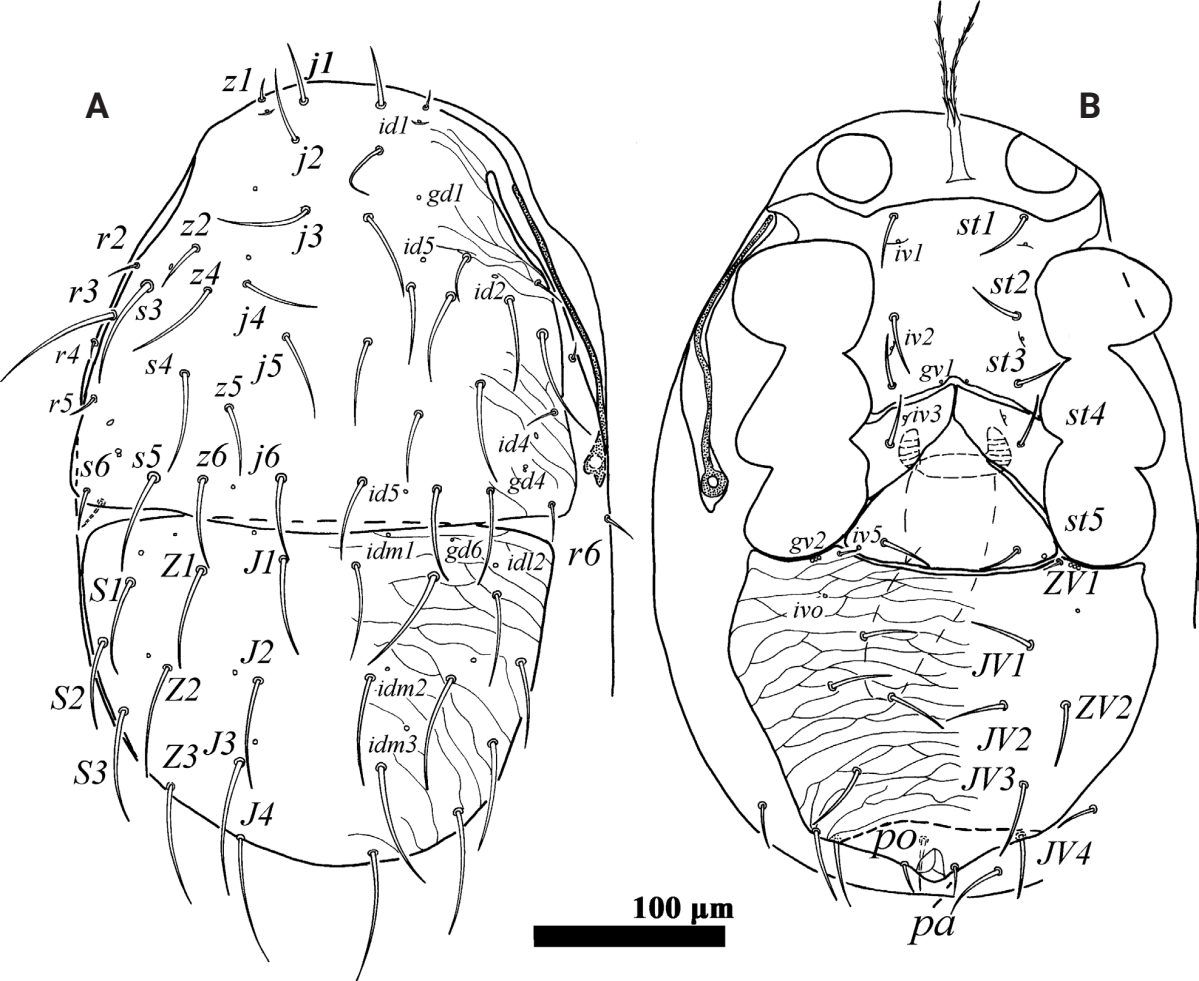
*Psilogamasus decemtrichus* Ren & Yi, sp. nov., female. A. Dorsum; B. Venter.

***Dorsum*** (Fig. 6A). Podonotal and opisthonotal shields separated and with rough reticulation. Podonotal region with 19 pairs of setae, of which 18 pairs on podonotal shield (seta *r5* on podonotal shield) and two pairs (setae *r4* and *r6*) off shield. Opisthonotal shield bearing ten pairs of setae. All dorsal setae smooth, setae *z1*, *s6*, *r2, r4*, *r5* and *r6* tiny. Lengths of dorsal setae: *j1* 28–30, *j2* 35–38, *j3* 47–49, *j4* 39–41, *j5* 47–49, *j6* 40–42, *z1* 7–8, *z2* 27–28, *z4* 47–50, *z5* 90–95, *z6* 57–62, *s3* 31–34, *s4* 58–61, *s5* 60–62, *s6* 15–17, *r2* 15–17, *r3* 159–165, *r4* 15–17, *r5* 16–17, *r6* 17–18, *J1* 50–54, *J2* 56–57, *J3* 63–65, *J4* 55–59, *Z1* 50–56, *Z2* 62–65, *Z3* 58–59, *S1* 48–52, *S2* 48–50, *S3* 58–60.

**Figure 7.**
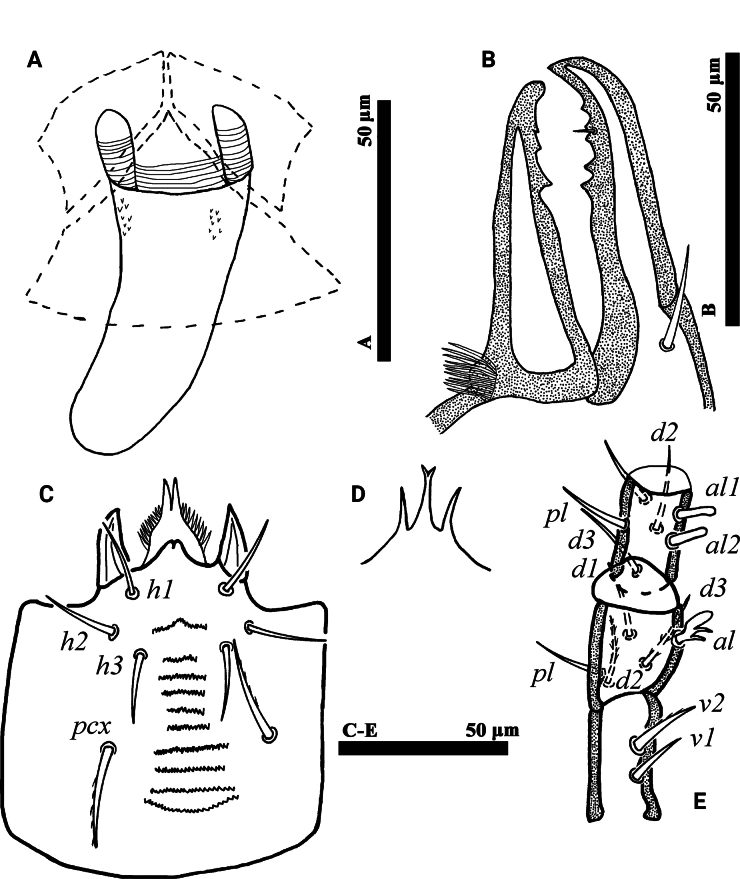
*Psilogamasus decemtrichus* Ren & Yi, sp. nov., female. A. Endogynium; B. Chelicera; C. Subcapitulum; D. Gnathotectum; E. Trochanter, femur and genu of palp.

***Venter*** (Figs 6B, 7A). Tritosternum with two pilose laciniae (64–73) and a smooth base (24–29). Presternal platelets absent. Sternal shield without reticulation, bearing three pairs of setae (*st1*–*st3*), *st1* (66–72) longer than *st2* (58–64) and *st3* (46–50). Metasternal shields separated from sternal shield by medially arched groove, bearing setae *st4* (45–52). Epigynial shield bearing setae *st5* (35–37). Endogynium tongue-shaped with some small teeth on each side of the leading edge, and anterolaterally with two inner roller-shaped structures underneath metasternal shields, connected by a ribbon structure (Fig. 7A). Setae *ZV1* and gland pores *gv2* present. Opisthogastric shield reticulate, bearing six pairs of setae (*JV1*–*JV4*, *ZV1* and *ZV2*). Setae *pa* and *po* equal in length (17–23). Peritrematal groove length 356–379, extending anteriorly to level of coxa II. Opisthogastric soft cuticle with three pairs of setae. All ventral setae smooth. Lengths of setae on opisthogastric shield: *JV1* 42–45, *JV2* 46–49, *JV3* 52–56, *JV4* 47–48, *ZV1* 12–15, *ZV2* 45–48.

***Gnathosoma*** (Fig. 7B–E). Movable digit of chelicera with three teeth, fixed digit with five teeth (Fig. 7B). Corniculus length 23–28, short and horn-shaped; deutosternal groove with ten denticulate rows. Setae *h1*–*h3* smooth, *h1* 25–5026, *h2* 21–23, *h3* 22–25 in length; *pcx* slightly pilose, 32–36 in length (Fig. 7C). Gnathotectum (Fig. 7D) with three prongs, central one bifid apically and lateral ones acuminate distally, emerging from nude base. Palp length 224–232; trochanter with two setae, *v2* slightly pilose; femur with five setae, of which *al* comblike, *d3* pilose; genu with six setae, of which *al1* and *al2* spatulate distally, remaining setae simple. Trochanter, femur and genu of palp as in Fig. 7E.

**Figure 8.**
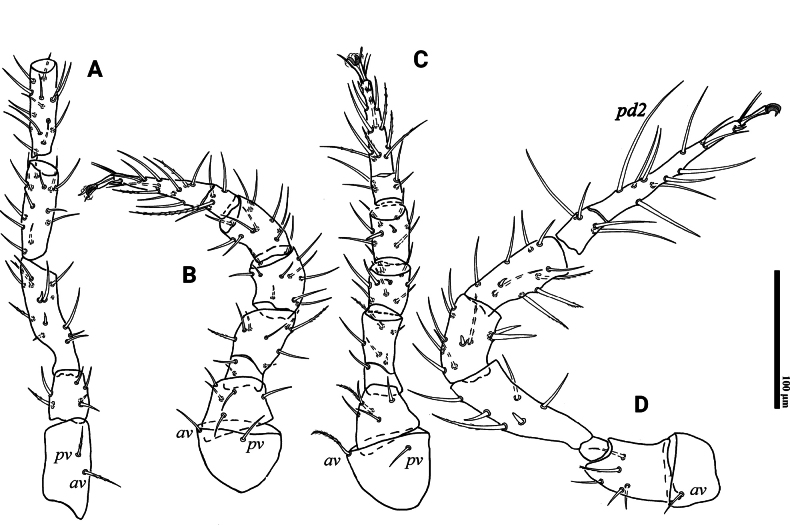
*Psilogamasus decemtrichus* Ren & Yi, sp. nov., female. A. Coxa–tibia of leg I; B. Leg II; C. Leg III; D. Leg IV.

***Legs*** (Fig. 8). Lengths of legs I–IV: I 470–481, II 294–326, III 305–320, IV 465–485. Most leg setae smooth. Setae *av1* and *pv1* on tarsi II–IV spur-like. Setae *pd2* on tarsus IV, length 95–107, longer than all other setae.

##### Other stages.

Unknown.

##### Etymology.

The name of the species is derived from “ decem-”, meaning “ten”, and–trichus, meaning “setae”. It refers to the opisthonotal shield with ten pairs of setae in females, which is diagnostic for this species.

##### Material examined.

***Holotype*.** China • 1 ♀ (slide no. YN2022070601), Yunnan Province, Pu’er City, Jingdong Yi Autonomous County, Taizhong Town; 24°32'28"N, 101°1'25"E; 2466 m a.s.l.; 6 Jun. 2022; collected from moss by Ye-Yi Shuai and Gui-Ming Mu. ***Paratypes*.** China • 4 ♀ (YN2022070602–YN2022070605), the same data as the holotype.

The holotype and paratypes are deposited in the Institute of Entomology, Guizhou University, Guivang, China (GUGC)

##### Remarks.

*Psilogamasus decemtrichus* Ren & Yi, sp. nov. can be easy distinguished from other species of the genus due to the setal number on the opisthonotal shield, which bears ten pairs rather than five or six pairs, and the presence of setae *r5*, *ZV1* and gland pores *gv2*.

#### ﻿ Psilogamasus bidens

Taxon classificationAnimaliaMesostigmataParasitidae

Ren & Yi sp. nov.

D4648A97-3F9A-5169-98EB-E15206E1FF21

https://zoobank.org/769DEA9D-2AD9-4DCC-80D6-3FEF7C974193

Figs 9, 10, 11

##### Diagnosis.

**Female.** Podonotal and opisthonotal shields with 18 and five pairs of setae, respectively; setae *j1* and *r3* slightly pilose; endogynium tongue-shaped with some small teeth on each side of the leading edge; opisthogastric shield bearing six pairs of setae; setae *ZV1* and gland pores *gv2* absent; Peritrematal groove extending anteriorly to beyond coxa II; movable digit of chelicera with four teeth.

##### Description.

**Female** (*N* = 4). Idiosoma weakly sclerotized, length 575–668, width 408–425.

**Figure 9.**
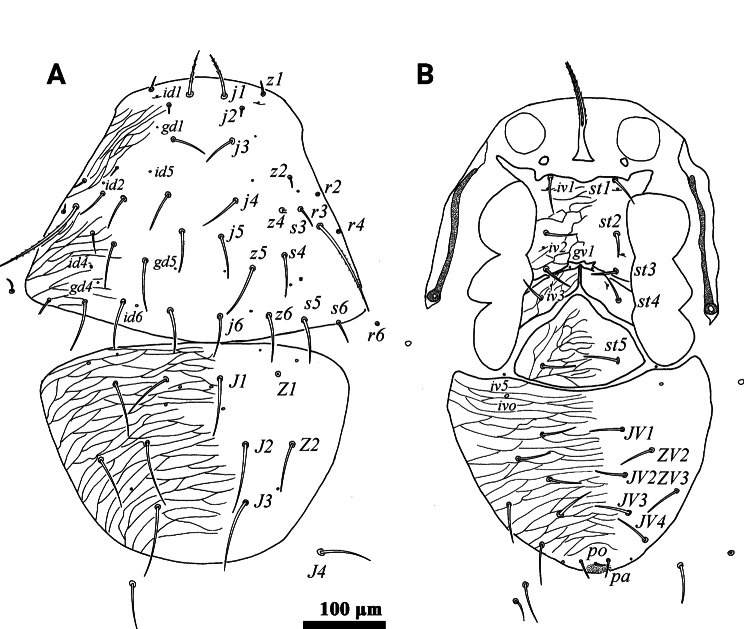
*Psilogamasus bidens* Ren & Yi, sp. nov., female. A. Dorsum; B. Venter.

***Dorsum*** (Fig. 9A). Podonotal and opisthonotal shields separated and with reticulation. Podonotal region with 19 pairs of setae, of which 18 pairs are on podonotal shield and a pair (seta *r6*) is off shield. Opisthonotal shield bearing five pairs of setae. Seta *J4* off shield. Setae *j1* and *r3* slightly pilose, *j2*, *z1*, *z2*, *r2, r4* and *r6* tiny. Lengths of dorsal setae: *j1* 50–55, *j2* 8–11, *j3* 42–46, *j4* 48–52, *j5* 53–55, *j6* 53–58, *z1* 13–18, *z2* 9–13, *z4* 47–50, *z5* 67–72, *z6* 63–65, *s3* 34–38, *s4* 53–56, *s5* 56–62, *s6* 16–20, *r2* 13–18, *r3* 113–121, *r4* 10–13, *J1* 66–70, *J2* 75–80, *J3* 77–82, *J4* 45–50, *Z1* 65–70, *Z2* 61–65.

**Figure 10.**
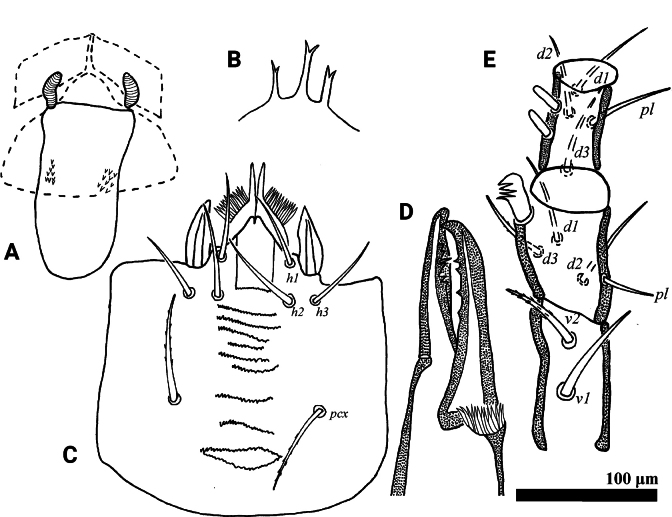
*Psilogamasus bidens* Ren & Yi, sp. nov., female. A. Endogynium; B. Gnathotectum; C. Subcapitulum; D. Chelicera; E. Trochanter, femur and genu of palp.

***Venter*** (Figs 9B, 10A). Tritosternum with two pilose laciniae and a smooth base. Pair of small presternal platelets present. Sternal shield with reticulation, bearing three pairs of setae (*st1*–*st3*), *st1* (42–46) longer than *st2* (34–37) and *st3* (33–37). Metasternal shields separated from sternal shield by medially arched groove, bearing setae *st4* (30–35). Epigynial shield bearing setae *st5* (37–43). Endogynium saccular, tongue-shaped in ventral view, with some small teeth on each side, and anterolaterally with two inner reniform structures (Fig. 10A). Setae *ZV1* and gland pores *gv2* absent. Opisthogastric shield reticulate, bearing six pairs of setae (*JV1*–*JV4*, *ZV2* and *ZV3*). Setae *pa* (22–25) and longer than *po* (15–17). Peritrematal groove length 178–188, extending to anterior level of coxa II. Peritrematal shield fused with podonotal shield anteriorly and separated at level of seta *j2*. Opisthogastric soft cuticle with two pairs of setae. All ventral setae smooth. Lengths of setae on opisthogastric shield: *JV1* 37–42, *JV2* 40–44, *JV3* 44–47, *JV4* 47–50, *ZV2* 47–51, *ZV3* 49–53.

***Gnathosoma*** (Fig. 10B–E). Gnathotectum (Fig. 2B) with three prongs, each apically bifid and emerging from nude base. Corniculus length 32–38, short and horn-shaped; deutosternal groove with ten denticulate rows. Setae *h1*–*h3* smooth, *h1* 52–58, *h2* 40–43, *h3* 57–63 in length; *pcx* pilose, 70–75 in length (Fig. 10C). Movable digit of chelicera with four teeth, fixed digit with five teeth (Fig. 10D). Palp trochanter bearing two setae, *v2* pilose; femur with five setae, of which *al* comblike; genu with six setae, of which *al1* and *al2* spatulate distally, remaining setae smooth. Trochanter, femur and genu of palp as in Fig. 10E.

**Figure 11.**
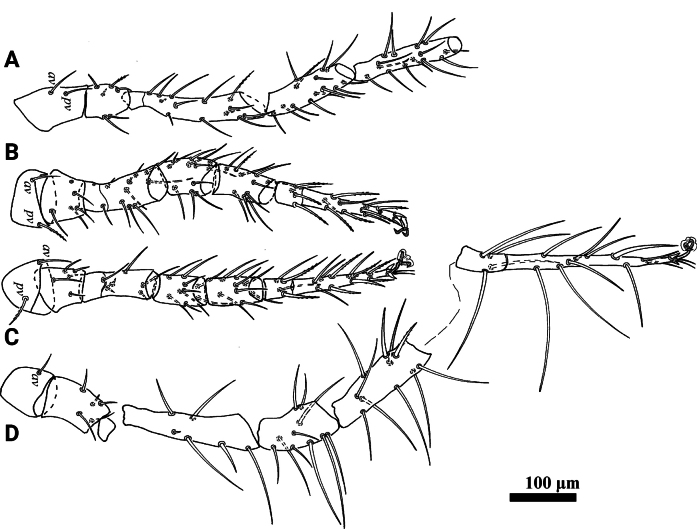
*Psilogamasus bidens* Ren & Yi, sp. nov., female. A. Coxa–tibia of leg I; B. Leg II; C. Leg III; D. Leg IV.

***Legs*** (Fig. 11). Lengths of legs I–IV: I 783–850, II 518–576, III 508–580, IV 937–970. Most leg setae smooth and setae on femur IV, genu IV, tibia IV and tarsi IV usually longer than setae on other legs. Most setae on tarsi II and tarsi III slightly pinnate. Setae *av1* and *pv1* on tarsi II–IV spur-like.

##### Other stages.

Unknown.

##### Etymology.

This new species is named based on the three prongs of the gnathotectum, where each prong exhibits an apical bifurcation (bidens).

##### Material examined.

***Holotype*.** China • 1 ♀ (slide no. YN2024051601), Yunnan Province, Honghe Hani and Yi Autonomous Prefecture, Pingbian County, Daweishan National Nature Reserve; 22°54'48"N, 103°41'47"E; 2038 m a.s.l.; 16 May, 2024; collected from decomposing leaves by Hu-Die He and Rong Ren. ***Paratypes*.** China • 1 ♀ (YN2024051602), the same data as the holotype. China • 1 ♀ (YN2024052901), Yunnan Province, Baoshan City, Longyang District, Mangkuan Yi and Dai Ethnic Township, Gaoligong Mountain National Nature Reserve; 25°17'28"N, 98°46'17"E; 2392 m a.s.l.; 29 May, 2024; collected from decomposing leaves by Hu-Die He and Rong Ren. China • 1 ♀ (YN2024051701), Yunnan Province, Honghe Hani and Yi Autonomous Prefecture, Mengzi City; 23°11'25"N, 103°22'54"E; 1649 m a.s.l.; 17 May, 2024; collected from moss by Hu-Die He and Rong Ren.

The holotype and paratypes are deposited in the Institute of Entomology, Guizhou University, Guivang, China (GUGC)

##### Remarks.

The female of the newly described species is morphologically similar to *P. longascidiformis* in the setal number on the opisthonotal shield, dental number of the fixed digit on chelicera, and the endogynium with a tongue-shaped suck. However, the female *P. bidens* Ren & Yi, sp. nov. is different from *P. longascidiformis* as follows: (1) opisthogastric shield bearing six pairs of setae, vs. five pairs in the latter; (2) dorsal setae *j1* and *r3* pilose, vs. simple in the latter; (3) fixed digit of chelicera with four teeth, vs. three in the latter; and (4) setae *j3* about four times longer than *j2* in length, vs. equal in the latter.

## ﻿Discussion

The placement of the species *Parasitus truncatus* Tseng, 1995 needs some discussion. This species was known only from the adult females collected from litter in Taiwan province, China (Tseng 1995). The genus *Parasitus* was established by Latreille in 1795 with *Parasitus coleoptratorum* Latreille, 1758 as its type species (Latreille 1795). The main characteristics of females of this genus are the separated podonotal and opisthonotal shields; dorsum with more than 40 pairs of setae; setae *z5* of dorsal hexagon markedly different in form from *j5* and *j6*, usually thicker, long and pilose distally; opisthogastric shield bearing rarely more than 30 pairs of setae; seta *al* of palp femur bifid, and setae *al1* and *al2* of palp genu entire, setiform or spatulate distally (Hyatt 1980). *Parasitus truncatus* is different from the mites of *Parasitus*, especially the type species *P. coleoptratorum*, in the following characteristics: dorsum with 22 pairs of setae; setae *z5* similar to *j5* and *j6* in form (smooth); opisthogastric shield bearing five pairs of setae; setae *ZV1* and gland pores *gv2* absent; seta *al* of palp femur comblike (Tseng 1995). The common features of female *P. truncatus* with *Psilogamasus* are: dorsum with less than 30 pairs of setae, which of podonotal shield with 16–18 pairs of setae and opisthonotal shield with five to six pairs of setae (ten pairs only *P. decemtrichus*); setae *z5* similar to *j5* and *j6* in form (smooth); opisthogastric shield bearing five to six pairs of setae; setae *ZV1* and gland pores *gv2* absent (present only in *P. decemtrichus* sp. nov.); seta *al* of palp femur comblike, *al1* and *al2* of palp genu entire and spatulate distally; peritrematal shields free posteriorly; and peritrematal groove extending to anterior level of coxa II (Tseng 1995; Yao et al. 2020). Moreover, the endogynium of *P. truncatus* has a long, tongue-shaped structure, which is similar to those of six species (namely, *P. decemtrichus* sp. nov., *P. hurlbutti*, *P. lingulatus*, *P. longascidiformis*, *P. pentatideus* and *P. bidens* sp. nov.) in *Psilogamasus*. Furthermore, Table 1 compares the main morphological features of *P. truncatus* with those of the six species.

**Table 1.** Morphological variations in females of *Psilogamasus decemtrichus* Ren & Yi, sp. nov., *Psilogamasus hurlbutti* (Athias-Henriot, 1969), *Psilogamasus lingulatus* (Bai & Ma, 2013), *Psilogamasus longascidiformis* (Ma & Lin, 2005), *Psilogamasus pentatideus* (Yao & Jin, 2020), *Psilogamasus bidens* Ren & Yi, sp. nov. and *Psilogamasus truncatus* (Tseng, 1995), comb. nov.

Mite	*P. decemtrichus* sp. nov.	*P. hurlbutti*	*P. lingulatus*	*P. longascidiformis*	*P. pentatideus*	*P. bidens* sp. nov.	*P. truncatus* comb. nov.
Setal number on podonotal shield	18 pairs	18 pairs	16–18 pairs	17 pairs	18 pairs	18 pairs	16 pairs
Setal number on opisthonotal shield	10 pairs	6 pairs	6 pairs	5 pairs	5 pairs	5 pairs	6 pairs
Setal number on opisthogastric shield	6 pairs	5 pairs	5 pairs	5 pairs	5 pairs	6 pairs	5 pairs
Dental numbers of movable digit on chelicera	3	4	4	3	4	4	4
Dental numbers of fixed digit on chelicera	5	7	5	5	7	5	9
Shape of setae *j1*	smooth	smooth	pilose	smooth	smooth	pilose	smooth
Base of gnathotectum	nude	nude	denticles	denticles	denticles	nude	denticles
Small teeth in endogynium	present	present	present	absent	present	present	absent

Therefore, we conclude that *P. truncatus* must be included in *Psilogamasus*.

### ﻿*Psilogamasus truncatus* (Tseng, 1995), comb. nov.

**Original designation.***Parasitus truncatus* Tseng, 1995: 32.

The mites of *Psilogamasus* occur in litter, moss, decomposing leaves, soil and humus. The geographical distribution of *Psilogamasus* has, until now, been limited to Tanzania, America, and China. This limited distribution may be related to the host species, collection times, and collection methods (Athias-Henriot 1969; Hennessey and Farrier 1989; Tseng 1995; Ma and Lin 2005; Yao et al. 2020). Therefore, in future taxonomic studies, more attention should be paid to host ranges and the use of diverse collection methods over different periods to better understand their geographical distribution.

### ﻿Key to adults of *Psilogamasus* species (updated from Yao et al. (2020))

**Table:** 

**Females**
1	Opisthonotal shield with ten pairs of setae; setae *ZV1* and gland pores *gv2* present	***P. decemtrichus* sp. nov.**
–	Opisthonotal shield with five pairs of setae; setae *ZV1* and gland pores *gv2* absent	**2**
2	Opisthonotal shield with six pairs of setae	**3**
–	Opisthonotal shield with five pairs of setae	**5**
3	Dorsal setae *j1* pilose	***P. lingulatus***
–	Dorsal setae *j1* smooth	**4**
4	Endogynium with small teeth; base of gnathotectum nude	***P. truncatus* comb. nov.**
–	Endogynium without teeth; base of gnathotectum denticular	***P. hurlbutti***
5	Peritrematal groove extending anteriorly to posterior of coxa III; movable digit of chelicera with three teeth	**6**
–	Peritrematal groove extending anteriorly to beyond coxa II; movable digit of chelicera with four teeth	**7**
6	Podonotal shield with 18 pairs of setae; the base of epistome smooth; epigynium serrate on both sides	***P. brachysternalis***
–	Podonotal shield with 17 pairs of setae; the base of epistome with small denticles; epigynium smooth on both sides	***P. longascidiformis***
7	Opisthogastric shield with six pairs of setae	**8**
–	Opisthogastric shield with five pairs of setae	***P. pentatideus***
8	Podonotal shield with 16 pairs of setae; dorsal setae *j1* smooth	***P. pentasetosus***
–	Podonotal shield with 18 pairs of setae; dorsal setae *j1* pilose	**9**
9	Dorsal setae *r3* smooth; endogynium with a short semi-circular sac structure	***P. brachysaccatus* sp. nov.**
–	Dorsal setae *r3* pilose; endogynium with a long tongue-shaped structure	***P. bidens* sp. nov.**
**Males**
1	Dorsal setae *j1* pilose; gnathotectum emerging from nude base and lateral prongs acuminate distally	***P. brachysaccatus* sp. nov.**
–	Dorsal setae *j1* simple; gnathotectum emerging from small teeth base and lateral prongs split distally	**2**
2	Dorsal seta on fixed digit of chelicera smooth; central prong of gnathotectum apically divided into 2–3 branches	***P. pentatideus***
–	Dorsal seta on fixed digit of chelicera forked; central prong of gnathotectum serrated apically	***P. hurlbutti***

## Supplementary Material

XML Treatment for Psilogamasus

XML Treatment for Psilogamasus brachysaccatus

XML Treatment for Psilogamasus decemtrichus

XML Treatment for Psilogamasus bidens

## ﻿Additional information

### Use of AI

No use of AI was reported.

### Author ORCIDs

Mao-Yuan Yao https://orcid.org/0000-0002-5475-046X

Jian-Xin Chen https://orcid.org/0000-0001-8787-6678

You-Fang Wu https://orcid.org/0000-0001-7968-5345

Rong Ren https://orcid.org/0009-0003-5331-3887

Tian-Ci Yi https://orcid.org/0000-0002-9953-3709

Dao-Chao Jin https://orcid.org/0000-0003-2727-5621

### Data availability

All of the data that support the findings of this study are available in the main text.
